# Supplementary material for: Cemiplimab for Locally Advanced and Metastatic Cutaneous Squamous-Cell Carcinomas: Real-Life Experience from the French CAREPI Study Group
Source: Cancers (Basel). 2021 Jul 15;13(14):3547. doi: 10.3390/cancers13143547 (PMC8305372; doi:10.3390/cancers13143547)
Supplement: Supplementary file 1 [file cancers-13-03547-s001.zip › cancers-1270524-supplementary.pdf]

Figure S1

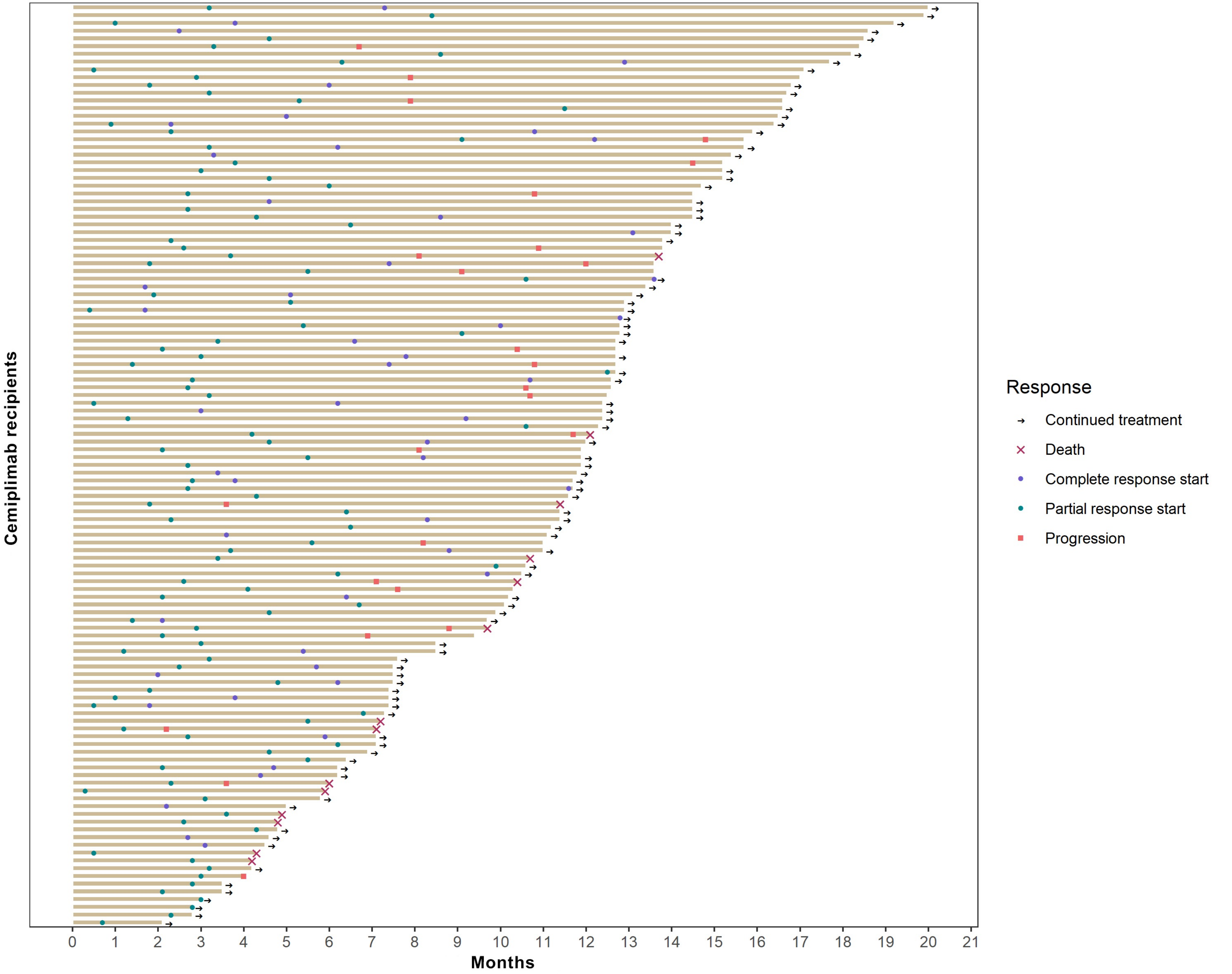
Cemiplimab recipients
0 1 2 3 4 5 6 7 8 9 10 11 12 13 14 15 16 17 18 19 20 21
Months
Response
Continued treatment
Death
Complete response start
Partial response start
Progression

Table S1: Therapies given to the 245 intent-to-treat patients before cemiplimab

**Prior Treatments**	**Value**
Radiotherapy	144 (60)
Surgery	193 (79)
Previous systemic treatment	121 (49)
Pre-cemiplimab line(s) of systemic treatment(s)/121	
1	93 (77)
2	19 (16)
3	8 (7)
4	1 (0.8)
Median duration of systemic treatment, months (range)	4 (0–65)
Systemic treatments administered/121	
Single chemotherapy	9 (7)
Combined chemotherapies	29 (24)
Chemotherapy plus radiotherapy	15 (12)
Anti-EGFR antibody	31 (26)
Anti-EGFR antibody plus radiotherapy	9 (7)
Anti-EGFR antibody plus chemotherapy	46 (38)
Anti PD-1	5 (4)
Others[a]	7 (6)

Results are expressed as number (%), unless stated otherwise.
EGFR, epidermal growth factor receptor; PD-1, programed cell-death protein-1
a Other treatments included: intratumoral bleomycin, 2; 1 each: combination chemotherapy, anti-EGFR and radiotherapy, anti-EGFR and imiquimod, vismodegib, paclitaxel and interferon followed by sorafenib, or unknown.
